# Assembly of a Rieske non-heme iron oxygenase multicomponent system from *Phenylobacterium immobile* E DSM 1986 enables pyrazon *cis*-dihydroxylation in *E. coli*

**DOI:** 10.1007/s00253-021-11129-w

**Published:** 2021-02-13

**Authors:** Andreas Hunold, Wendy Escobedo-Hinojosa, Elsa Potoudis, Daniela Resende, Theresa Farr, Per-Olof Syrén, Bernhard Hauer

**Affiliations:** 1 Institute of Biochemistry and Technical Biochemistry, Department of Technical Biochemistry, University of Stuttgart, Allmandring 31, 70569 Stuttgart, Germany; 2 School of Chemical Science and Engineering, Division of Applied Physical Chemistry, KTH Royal Institute of Technology, 100 44 Stockholm, Sweden; 3 Science for Life Laboratory, KTH Royal Institute of Technology, 171 21 Stockholm, Sweden

**Keywords:** *Phenylobacterium immobile*, Rieske non-heme iron oxygenases, Pyrazon oxygenase, *Cis*-dihydroxylation, Biodegradation, Biocatalysis

## Abstract

**Abstract:**

*Phenylobacterium immobile* strain E is a soil bacterium with a striking metabolism relying on xenobiotics, such as the herbicide pyrazon, as sole carbon source instead of more bioavailable molecules. Pyrazon is a heterocyclic aromatic compound of environmental concern and its biodegradation pathway has only been reported in *P. immobile*. The multicomponent pyrazon oxygenase (PPO), a Rieske non-heme iron oxygenase, incorporates molecular oxygen at the 2,3 position of the pyrazon phenyl moiety as first step of degradation, generating a *cis*-dihydrodiendiol. The aim of this work was to identify the genes encoding for each one of the PPO components and enable their functional assembly in *Escherichia coli*. *P. immobile* strain E genome sequencing revealed genes encoding for RO components, such as ferredoxin-, reductase-, α- and β-subunits of an oxygenase. Though, *P. immobile* E displays three prominent differences with respect to the ROs currently characterized: (1) an operon-like organization for PPO is absent, (2) all the elements are randomly scattered in its DNA, (3) not only one, but 19 different α-subunits are encoded in its genome. Herein, we report the identification of the PPO components involved in pyrazon *cis*-dihydroxylation in *P. immobile,* its appropriate assembly, and its functional reconstitution in *E. coli*. Our results contributes with the essential missing pieces to complete the overall elucidation of the PPO from *P. immobile*.

**Key points:**

*• Phenylobacterium immobile E DSM 1986 harbors the only described pyrazon oxygenase (PPO).*

*• We elucidated the genes encoding for all PPO components.*

*• Heterologous expression of PPO enabled pyrazon dihydroxylation in E. coli JW5510.*

**Supplementary Information:**

The online version contains supplementary material available at 10.1007/s00253-021-11129-w.

## Introduction

The most peculiar characteristic of *Phenylobacterium immobile* E DSM 1986 (from here referred as *P. immobile*) is its high nutritional specialization. This bacterium is fascinating for its extremely limited nutritional spectrum, since all isolated and described strains utilize synthetic aromatic compounds like pyrazon or phenazone as carbon source. Extraordinarily, *P. immobile* does not grow on common carbon sources like sugar alcohols, carboxylic acids (except succinic acid), amino acids (except phenylalanine), or on routine complex media (Lingens et al. 1985). *P. immobile* is the only characterized microorganism described to be capable of degrading pyrazon (Lingens et al. 1985). The herbicide pyrazon [5-amino-4-chloro-2-phenyl-3(2H)-pyridazinone] is a heterocyclic aromatic compound used since 1960, which inhibits photosynthesis and is used for the control of annual broad-leaved weeds, and on sugar beets. *P. immobile* strain E was isolated from soil samples obtained in Ecuador using a selection pressure strategy, employing pyrazon as sole carbon source (Engvild and Jensen 1969; Fröhner et al. 1970). The striking ability to use synthetic compounds as sole carbon source, while showing no growth on complex media, encouraged several studies in the 1970s, in which the pyrazon degradation by *P. immobile* was investigated by metabolic and enzymatic studies (Fröhner et al. 1970; Sauber et al. 1977b; Müller and Lingens 1980; Kreis et al. 1981; Schmitt et al. 1984). However, insights into how this catabolic ability was acquired and genetically encoded were not obtained.

**Fig. 1:**
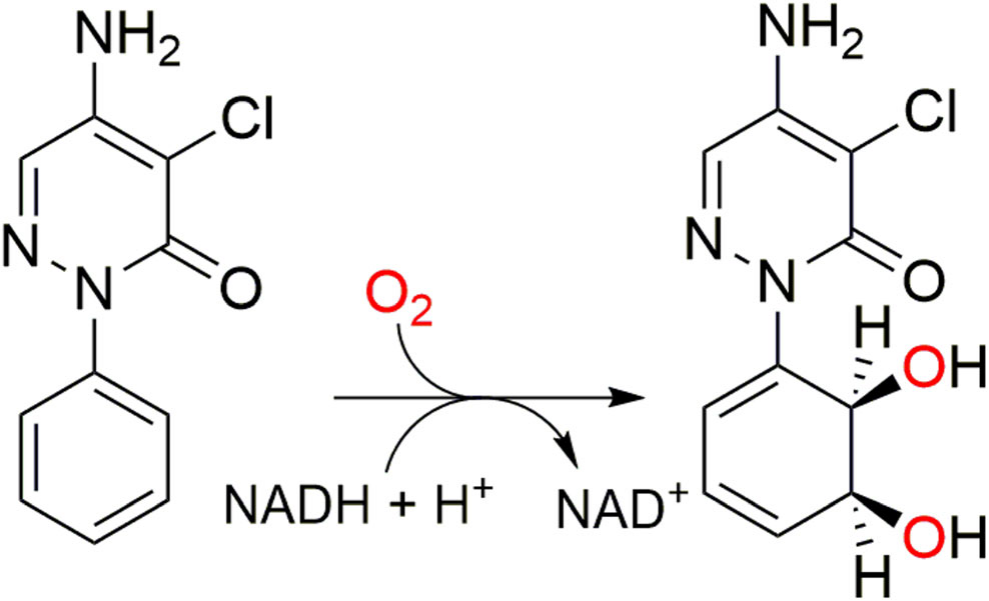
Dihydroxylation of pyrazon. The degradation of pyrazon (**a**) is initiated by the oxidation of its phenyl moiety to the corresponding *cis*-dihydrodihydroxy compound (**b**). The proposed reaction involves the pyrazon oxygenase, oxygen as co-substrate, and NADH**+**H^**+**^ as electron donor

The degradation of pyrazon is initiated by the oxidation of its phenyl moiety to the corresponding *cis-*dihydrodiendiol, activating the primary molecule for further degradation (Fig. 1) (Lingens et al. 1985). The responsible enzymatic system was purified and characterized (Sauber et al. 1977a). The results showed that three soluble protein components catalyze the enzymatic oxidation of pyrazon: (1) a flavoprotein component, which accepts the co-substrate NADH and functions as a ferredoxin-reductase; (2) a ferredoxin component as an electron carrier; and (3) an oxygenase component. The oxygenase catalyzes the dihydroxylation of two non-activated carbon atoms of the aromatic nucleus by the incorporation of molecular oxygen. It was assumed that the pyrazon oxygenase (PPO) resembled the three-component system reported for Rieske non-heme iron oxygenases (ROs). They consist of a flavoprotein reductase, an iron-sulfur ferredoxin, and a terminal iron-sulfur oxygenase (see Fig. S1) (Mason and Cammack 1992). The terminal oxygenase can be either a homo- (α_3_ or α_6_) or a heteromeric oligostructure (α_3_β_3_). The larger α-subunit is catalytically active and the smaller β-subunit has only a structural purpose in the systems characterized so far. The α-subunit has a Rieske [2Fe-2S] cluster domain, which accepts the electrons from the ferredoxin and passes them *via* an aspartate to the mononuclear iron of the neighboring α-subunit for catalysis (Ferraro et al. 2005). In several characterized ROs, the components are organized as a gene cluster either in the genomic DNA or on a catabolic plasmid of the cell, along with other enzymes involved in the oxidative upper pathway (Batie et al. 1991; Werlen et al. 1996; Nam et al. 2001). In 2015, the genome of *P. immobile* strain E DSM 1986 was sequenced (Reznicek et al. 2015). A targeted sequence analysis revealed an outstanding number of 19 different encoded catalytic α-subunits of ROs, surpassing any biological system presently described. Another remarkable genetic feature of this strain is the lack of gene clusters frequently observed for ROs. None of the α-subunits were clustered with genes encoding either electron transport proteins, or β-subunits. In addition, the strain harbors one plasmid encoding two of the 19 α-subunits, but none of the genes encoding genes for other RO components. Despite all knowledge regarding the distinctive enzymatic properties of PPO, the genes encoding for this highly interesting RO remained unknown due to the lack of molecular tools in the past. Although, there are still not available customized molecular tools for *P. immobile*, it has been shown that employing *E. coli* as an expression host is a useful resource, even in cases aiming for the heterologous expression of proteins from bacteria presenting large phylogenetic distances with *E. coli* (Rosano and Ceccarelli 2014). The aims of this work were (1) to identify the genes encoding each one of the PPO components in *P. immobile* and (2) to enable the functional assembly of the system for pyrazon *cis*-dihydroxylation in *E. coli*. Our findings contribute the essential missing pieces for the whole elucidation of the PPO system from *P. immobile*. This work expands the portfolio of ROs and enables further studies for the bioremediation of pyrazon and other related hazardous, synthetic compounds. Furthermore, we provide the foundations for a potential biotechnological application of PPO, addressing the biocatalytic generation of valuable vicinal *cis*-dihydrodiendiols, which are important building blocks in the pharmaceutical and chemical industry (Hudlicky et al. 1999; Gally et al. 2015; Halder et al. 2018).

## Methods

### Culture conditions

*Phenylobacterium immobile* strain E (DSM 1986; ATCC® 35973) cultures were routinely grown at 30 °C, 180 rpm, in minimal medium B and 1% (w/v) phenazone, as described by Lingens *et al.* (Lingens et al. 1985). The purity of the culture was checked regularly by microscopy, along with axenicity controls grown on LB medium. To prepare culture plates, 2% (w/v) agar was added to the medium before sterilization. The strain was stored as 25% (w/v) glycerol stocks at −80 °C. *Escherichia coli* XL-1 blue and JW5510 were grown at 37 °C in LB medium. When required, antibiotics were added to *E. coli* cultures in the following concentrations: 100 μg/mL ampicillin and 30 μg/mL chloramphenicol. A list of used organisms and their features is given in the supplementary information (see Table S1).

### Targeted proteome investigations

The proteome of *P. immobile* was examined after growth in any of the following carbon sources: pyrazon, phenazone and L-phenylalanine. *P. immobile* was streaked out from glycerol stock on minimal medium B agar plates, containing 0.1% (w/v) phenazone, L-phenylalanine, or pyrazon). Freshly grown agar plates were used to inoculate 200 mL shaking flasks, containing 40 mL of minimal medium B supplemented with 0.1% (w/v) of either phenazone, L-phenylalanine, or 0.04% (w/v) of pyrazon to an OD_600_ of 0.1. The cultures were incubated at 30 °C and 180 rpm until the stationary phase (OD_600_ = 0.6) was reached. Each culture condition was sub-cultured three times. After reaching the stationary phase, in the third passage, cells were harvested (5000 x g, 20 min, 4 °C). The cell pellet was resuspended in KPi buffer (50 mM, pH 7.0) and concentrated to 1/80 with respect to the initial volume. Cells were disrupted by sonification (6 min; output 1-2; duty circle: 35%) (Sonifier B250, Branson, USA). The cell debris was centrifuged (40 min, 50000 x g, 4 °C) and resuspended in KPi buffer (50 mM, pH 7.0). Protein concentration (lysate and resuspended cell debris) was determined by the bicinchoninic acid test (Smith et al. 1985). For each sample, 50 μg of total protein was loaded to a CN-PAGE.

### CN-PAGE

The CN-PAGE was carried out according to the manufacturer’s specifications (SERVAGel™ N Native Gel Starter Kit, Serva, Heidelberg, DE).

### Proteome analysis

The proteome analysis was performed by mass spectrometry at the Core Facility Hohenheim Module Mass Spectrometry (University of Hohenheim, DE) with an EASY-nLC 1200 System coupled to a Q-Extractive mass spectrometer (Thermo Fisher Scientific, DE). Detected peptides were matched with the protein sequences of the annotated α-subunits by the software Mascot 2.6 (Matrix Science, UK) and transferred to the Scaffold Software 4.8.6 (Proteome Software, USA). All cultivations and proteome analyses were performed in biological triplicates.

### *P. immobile* DNA isolation

*P. immobile* cells were grown in minimal medium B, containing 0.1% (w/v) phenazone in a volume of 50 mL at 30 °C until stationary phase was reached. Culture was concentrated to 1/100 of the previous volume, and cells were disrupted by heat (80 °C, 10 min). The cell debris was centrifuged (40 min, 5,000 x g, 4 °C) and DNA concentration in the supernatant was measured by NanoDrop (ND-1000-Spektrometer, Thermo Fisher Scientific, Waltham, US).

### Cloning

All α- and β-subunit genes, as well as the redox partner genes, were cloned into pBAD33 or pBAD18 (Guzman et al. 1995) vector, respectively, using the isothermal DNA assembly according to Gibson (Gibson 2011). A list of the constructed plasmids is given in Table 1. Inserted genes were amplified by PCR from the previously isolated *P. immobile* DNA, using the KOD HS DNA polymerase (Novagene Inc., Madison, WI, USA), under the recommended conditions by the manufacturer. Plasmid backbones pBAD18 and pBAD33 were amplified accordingly (see also Table S2). Employed primer list is provided in the supplementary information (Table S3). PCR products were assessed by agarose electrophoresis (Lee et al. 2012). Amplified fragments were cut from agarose gel (0.7 %) for further purification. DNA fragment purification was done using the commercial kit Zymoclean Gel DNA Recovery Kit (Zymo Research Corp., Freiburg, DE), according to manufacturer’s instructions. Purified DNA was eluted with 20 μl ddH_2_O. Gibson one-step ISO assembly was done as described by Gibson et al*.* (Gibson 2011), and 1 μl of the reaction mixture was directly transformed into chemocompetent *E. coli* XL-1 blue cells by heat shock method (Froger and Hall 2007). Plasmids were isolated by alkaline lysis using the Zippy Plasmid Miniprep Kit (Zymo Research Corp., Freiburg, DE) according to manufacturer’s specifications. DNA sequence for all cloned fragments was verified by Sanger sequencing (GATC Biotech AG, Konstanz, Germany).

**Table 1:** List of constructed plasmids in this study

#	Plasmid	Size [bp]	Insert
1	pBAD18_ppoa2_ppob1	6012	reductase *ppoa2* + ferredoxin *ppob1*
2	pBAD18_ppoa1_ppob2	6012	reductase *ppoa1* + ferredoxin *ppob2* +
3	pBAD33_ppoc2	7621	α-subunit *ppoc2* + β-subunit *ppod*
4	pBAD33_ppoc3	7105	α-subunit *ppoc3*+ β-subunit *ppod*
5	pBAD33_ppoc4	7345	α-subunit *ppoc4*+ β-subunit *ppod*
6	pBAD33_ppoc5	7282	α-subunit *ppoc5*+ β-subunit *ppod*
7	pBAD33_ppoc6	7285	α-subunit *ppoc6*+ β-subunit *ppod*
8	pBAD33_ppoc7	7342	α-subunit *ppoc7*+ β-subunit *ppod*
9	pBAD33_ppoc8	7030	α-subunit *ppoc8*+ β-subunit *ppod*
10	pBAD33_ppoc9	7330	α-subunit *ppoc9*+ β-subunit *ppod*
11	pBAD33_ppoc10	7333	α-subunit *ppoc10*+ β-subunit *ppod*
12	pBAD33_ppoc11	7339	α-subunit *ppoc11* + β-subunit *ppod*
13	pBAD33_ppoc12	7336	α-subunit *ppoc12*+ β-subunit *ppod*
14	pBAD33_ppoc13	7321	α-subunit *ppoc13* + β-subunit *ppod*
15	pBAD33_ppoc14	7324	α-subunit *ppoc14*+ β-subunit *ppod*
16	pBAD33_ppoc15	7324	α-subunit *ppoc15*+ β-subunit *ppod*
17	pBAD33_ppoc16	7255	α-subunit *ppoc16*+ β-subunit *ppod*
18	pBAD33_ppoc17	7315	α-subunit *ppoc17*+ β-subunit *ppod*
19	pBAD33_ppoc18	7327	α-subunit *ppocc18*+ β-subunit *ppod*
20	pBAD33_alpha	6740	α-subunit *ppoc11*
21	pBAD33_beta	5855	β-subunit *ppod*

### Expression

In order to introduce any of the combinations of the redox partners, chemocompetent *E. coli* JW5510 cells were first transformed with either plasmid 1 or 2 by the heat shock method (Froger and Hall 2007). Chemocompetent *E. coli* JW5510 cells harboring plasmid 1 or 2 were retransformed with each one of the plasmids 3-19, containing the genes encoding for the α- and β-subunit(s). After the transformation steps, cells were plated on selective LB agar plates (100 μg/mL ampicillin, 30 μg/mL chloramphenicol) and incubated overnight at 37 °C. Liquid LB medium (5 mL) was inoculated with a single colony and cultivated overnight at 37 °C and 180 rpm in an incubation shaker (Infors AG, Bottmingen, CH). 2 L Erlenmeyer flasks, containing TB medium (400 mL), were inoculated with 4 mL of the overnight culture and incubated at 37 °C and 100 rpm to an OD_600_ = 1.2. Expression was induced by the addition of 0.1% (w/v) arabinose. Additionally, FeSO_4_ to a final concentration of 0.5 mM was added to the medium. Temperature and shaking were decreased to 25 °C and 100 rpm, and incubation was performed for 20 h. Cells were harvested by centrifugation (4 °C, 8000 x g, 30 min). Freshly produced cells expressing candidate PPO components were directly used for in vivo biotransformations.

### Biotransformations with *E. coli*

To prepare resting cells, *E. coli* JW5510 expressing candidate PPO components were resuspended (0.2 g_cww_/mL) in KPi buffer (100 mM, pH 7.0) and supplemented with 30 mM glucose for cofactor regeneration. Biotransformations were performed in 20-mL headspace vials containing 1 mL of reaction volume. The substrate concentration was 2 mM for substrates classified in group A (pyrazon, phenazone, and L-phenylalanine) or 10 mM for substrates classified in group B (phenylacetic acid, phenylpropionic acid, and cinnamic acid). Biotransformations were incubated at 30 °C and 180 rpm for 24 h. Negative controls were performed with *E. coli* JW5510 cells transformed with the pBAD33 empty vector. Group A substrates were analyzed by HPLC, while the ones from group B were analyzed by GC as it is described in the supplementary information (see Table S4, Table S5).

### SDS-PAGE

The SDS-PAGE was carried out according to the manufacturer’s specifications (12% ExpressPlus^TM^ PAGE gel, GensScript, Piscataway, NJ, USA).

### Biotransformations with *P. immobile*

*P. immobile* resting cells (0.1g_cww_/mL) were prepared employing grown cultures in minimal medium B supplemented with 1% (w/v) phenazone, at 30 °C and 180 rpm, in KPi buffer (50 mM, pH 7.0). Group A substrates were added at 2-mM final concentration and incubated at 30 °C and 180 rpm. Biotransformations were stopped at different reaction times (5–60 min), until the metabolite has accumulated to the desired extent.

### Alignment

The alignment of the different α-subunits was carried out by a multiple sequence alignment with the program Clustal Omega, which uses the HHalign algorithm and its default settings (Madeira et al. 2019; URL: www.ebi.ac.uk/Tools/msa/clustalo/; as of June 2020). The free software Jalview (URL: www.jalview.org; as of June 2020) was used to graphically display the alignment.

### Homology model

The homology model was generated with the software YASARA (YASARA Biosciences GmbH, Vienna, AUT). This software independently searches the UniProt database for related sequences and uses them to search the PDB (Protein Data Bank) database for related protein structures. The protein structures found are used by YASARA as a template to create a hybrid model. The obtained protein models were visualized with PyMOL 1.3.

## Results

Genomic information regarding *P. immobile* was only recently available (Reznicek et al. 2015). In the past, this hampered the elucidation of the genes encoding for the PPO components, its assembly, and the establishment of a heterologous expression platform enabling pyrazon dihydroxylation. Our first task was to organize the genetic information and assign each candidate to an open reading frame for each putative PPO component.

### Gene nomenclature

Two different ferredoxin-reductases, two ferredoxins, a single β-subunit, and 19 different α-subunits were identified and listed. A simplified view of all relevant genetic information is provided in Table 2, showing position and orientation in the gene locus, gene name assignments, putative assigned function, protein name assignations, corresponding expressed protein size in kDa, and NCBI Accession ID.

**Table 2:** *P. immobile* genetic information and name assignations for genes and proteins for each putative PPO component

Gene locus	Gene name	Assigned function	Protein name	Size [kDa]	NCBI Accession ID
Scaffold 1: 180533-183956(-)	*ppoa1*	ferredoxin reductase	PpoA1	43.82	WP_091736606.1
Scaffold 1: 677147-677930(+)	*ppoa2*	ferredoxin reductase	PpoA2	30.11	WP_091737989.1
Scaffold 1: 679301-679622(+)	*ppob1*	ferredoxin	PpoB1	11.60	WP_091736609.1
Scaffold 1: 2613528-2614037(+)	*ppob2*	ferredoxin	PpoB2	12.69	WP_091741309.1
Scaffold 2: 204405-205587(-)	*ppoc1*	oxygenase (α-subunit)	PpoC1	43.27	WP_091743116.1
Scaffold 1: 3614-5012(-)	*ppoc2*	oxygenase (α-subunit)	PpoC2	50.66	WP_091734813.1
Scaffold 1: 82414-83542(-)	*ppoc3*	oxygenase (α-subunit)	PpoC3	43.03	WP_091735006.1
Scaffold 1: 485708-487022(+)	*ppoc4*	oxygenase (α-subunit)	PpoC4	52.39	WP_091736066.1
Scaffold 1: 497066-498403(+)	*ppoc5*	oxygenase (α-subunit)	PpoC5	50.19	WP_091736097.1
Scaffold 1: 831257-832595(-)	*ppoc6*	oxygenase (α-subunit)	PpoC6	50.10	WP_091736878.1
Scaffold 1: 83505-836427(+)	*ppoc7*	oxygenase (α-subunit)	PpoC7	51.08	WP_091736895.1
Scaffold 1: 905071-905854(+)	*ppoc8*	oxygenase (α-subunit)	PpoC8	41.07	WP_091737075.1
Scaffold 1: 1173154-1174537(+))	*ppoc9*	oxygenase (α-subunit)	PpoC9	51.56	WP_091737675.1
Scaffold 1: 1549070-1550456(+)	*ppoc10*	oxygenase (α-subunit)	PpoC10	51.22	WP_091738530.1
Scaffold 1: 1550566-1551958(+)	*ppoc11*	oxygenase (α-subunit)	PpoC11	51.92	WP_091738533.1
Scaffold 1: 1552131-1553520(+)	*ppoc12*	oxygenase (α-subunit)	PpoC12	51.37	WP_091738536.1
Scaffold 1: 1553712-1555089(+)	*ppoc13*	oxygenase (α-subunit)	PpoC13	51.12	WP_091738539.1
Scaffold 1: 1561383-1562760(+)	*ppoc14*	oxygenase (α-subunit)	PpoC14	51.50	WP_091738549.1
Scaffold 1: 1851075-1852452(-)	*ppoc15*	oxygenase (α-subunit)	PpoC15	51.50	WP_091739359.1
Scaffold 1: 1852575-1853916(-)	*ppoc16*	oxygenase (α-subunit)	PpoC16	48.88	WP_091739362.1
Scaffold 1: 2850081-2851449(+)	*ppoc17*	oxygenase (α-subunit)	PpoC17	51.08	WP_091741932.1
plasmidic DNA	*ppoc18*	oxygenase (α-subunit)	PpoC18	51.43	WP_091743344.1
plasmidic DNA	*ppoc19*	oxygenase (α-subunit)	PpoC19	19.84	WP_091737403.1
Scaffold 2: 207087-207633(-)	*ppod*	oxygenase (β-subunit)	PpoD	21.53	WP_091743119.1

### Proteome analysis of *P. immobile*

**Fig. 2:**
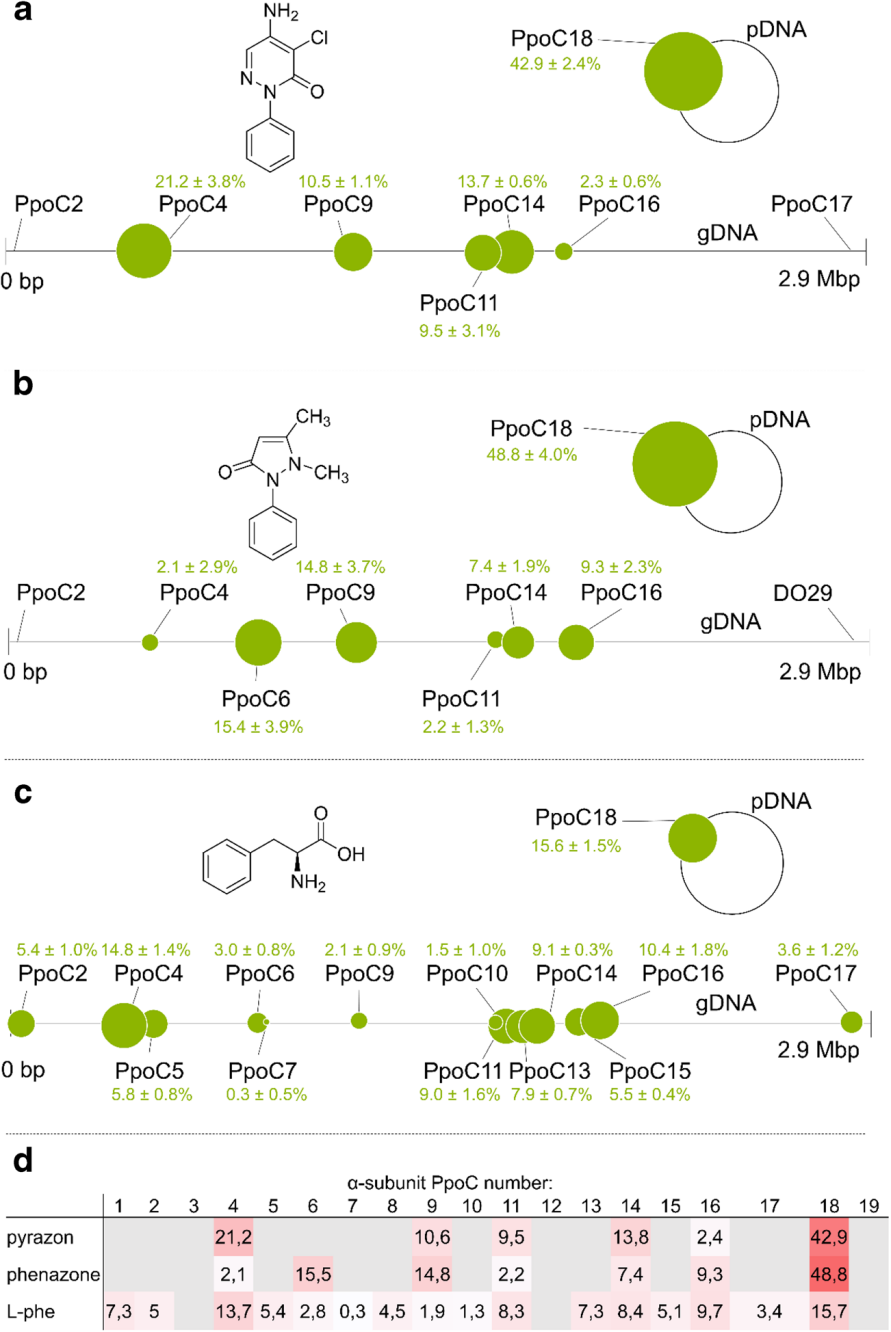
Targeted proteome analysis of *P. immobile*. Sole carbon sources: pyrazon (**a**), phenazone (**b**), and L-phenylalanine (**c**), were employed to determine the differential expression of the different 19 α-subunits. Gene loci of the detected α-subunits (*ppoc1* - *ppoc19*) and peptide distribution is provided. Different sizes in green dots aim to represent the peptide distribution (percentage). The locus is depicted as a linear structure for genomic DNA (**gDNA)** or as a circular structure for plasmidic DNA (**pDNA)**. The comparative heatmap (**d**), shows the expression ratio of the different α-subuinits among the tested carbon sources

To investigate how different carbon sources influence gene expression in *P. immobile*, we performed a targeted proteome analysis. Since *P. immobile* is a slow growing oligotrophic bacterium, it was only feasible to cultivate with certain carbon sources. Considering the limited carbon source scope of *P. immobile*, and the fact that liquid cultures with non-aromatic compounds are not viable, a proteome analysis under conditions lacking PPO expression was not possible. Therefore, we focused on the differential expression of the 19 α-subunits, when the bacterium is exposed to three different aromatic carbon sources. We selected the following carbon sources: pyrazon (*T*_*d*_ = 7.5 h), phenazone (*T*_*d*_ = 7 h), and L-phenylalanine (*T*_*d*_ = 8.5 h). To ensure a resilient growing adaptation to each carbon source, the bacterium was subcultivated three times starting from an OD_600_ = 0.1, until stationary phase was reached. We compared the peptide distribution depending on the carbon source. For this purpose, the number of peptides assigned to a single α-subunit (probability > 95%) was divided by the total number of all α-subunit peptides detected in the proteome. A graphical representation of the peptide distribution in percentage, for each α-subunit, and its gene loci is shown in Fig. 2.

Peptide assignations for 17 out of 19 different α-subunits were feasible with a probability of over 95% (raw data is provided in Fig. S2). As shown in Fig. 2, 16 of the gene loci are scattered over the largest scaffold_1 (2.9 Mb). The gene locus *ppoc1* is located on a smaller scaffold (269 kb, not shown), while gene locus *ppoc1*8 is on the plasmid. The analysis showed that six α-subunits (PpoC: 4, 9, 11, 14, 16, and 18) are permanently expressed, regardless of the carbon source employed, whereas in all cases, the expression of the α-subunits PpoC3, PpoC12, and PpoC19 was not detectable. Interestingly, the α-subunit encoded on the plasmid (*ppoc18*) is the one showing the strongest expression with all three carbon sources: 42.9 ± 2.4%, 48.7 ± 4.0%, and 15.7 ± 1.5 %, for pyrazon, phenazone, and L-phenylalanine, respectively. Pyrazon was the carbon source inducing the smallest number of α-subunits. The only additionally induced α-subunit with phenazone was PpoC6. However, L-phenylalanine was the carbon source that induced the highest number of α-subunits. Besides the similar expression profile of the six α-subunits observed for pyrazon and phenazone, the α-subunits PpoC, 1, 2, 5, 7, 8, 10, 13, 15, and 17, were also induced. In this case, the peptide distribution was moderately distributed among all expressed α-subunits. Nevertheless, PpoC18 was still the one showing the strongest expression, although not as high as with pyrazon and phenazone. To perform the targeted proteome analysis, cell proteins were first separated by CN-PAGE in order to facilitate our investigations regarding peptide distribution, but also to enable the electrophoretic separation without the denaturation of the quaternary protein structure. The resulting separation indicated a size ranging around 200 kDa for all oxygenases. It can be inferred that the quaternary conformation or the oxygenase component in PPO from *P. immobile* is rather a heteromeric structure (α_3_β_3_) than a homomeric one(α_3_), taking into account that the average size for α-subunits (P8poC1-PpoC19) is 51 kDa and 21.5 kDa for the β-subunit PpoD (Table 2).

### Activity reconstitution of PPO in *E. coli*

**Fig. 3:**
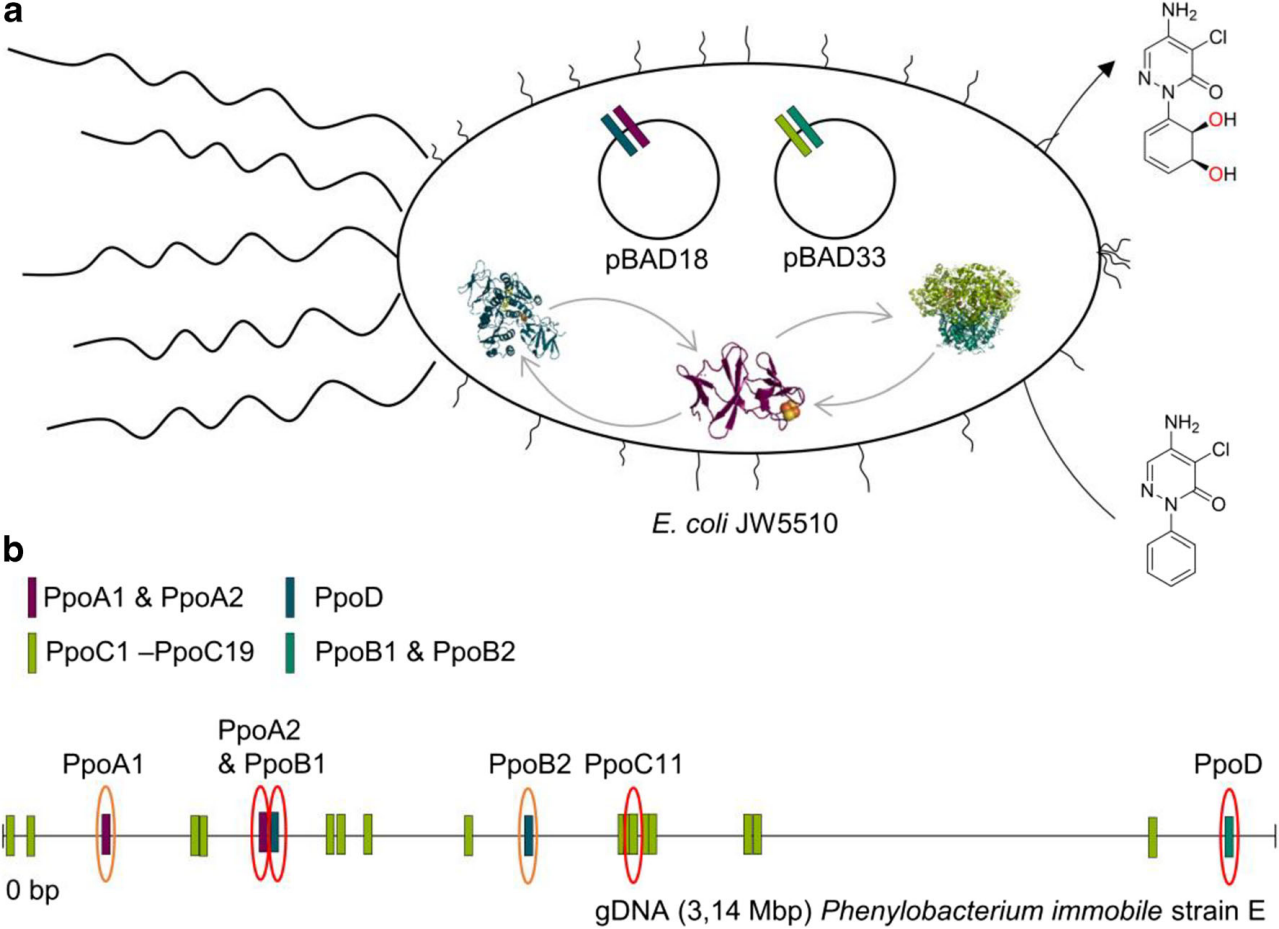
Cloning strategy: heterologous expression and assembly of the PPO components in *E. coli.* (**a**); color code assignations PpoA (violet), PpoB (blue), PpoC (green), and PpoD (cyan); schematic linear gDNA structure (**b**) intends to depict the locus of the identified components (in circles), enabling the functional assembly of PPO

So far, there are no molecular tools specifically customized for *P. immobile*. Therefore, our strategy was to employ *E. coli* JW5510 as a host for the heterologous expression of all candidate PPO components. The designed expression platform consisted of two different co-expressed plasmids. One harboring the redox partners (ferredoxin and ferredoxin-reductase) and another containing both α- and β-subunit encoding for the oxygenase components (Fig. 3a). To construct plasmids **3–19** (Table 1), genes *ppoc2*-*ppoc18* were amplified from *P. immobile* DNA and cloned together with the β-subunit gene *ppod* into the plasmid pBAD33. Since the ferredoxin gene *ppoa2* (Scaffold 1:677147-677930(+)) and the ferredoxin-reductase gene *ppob1* (Scaffold 1:679301-679622(+)) are positioned together in the genome, both were cloned into the vector pBAD18 to construct plasmid **1**. The other identified redox partners (genes *ppoa1* and *ppob2)*, distantly scattered in the scaffold, were also cloned into the vector pBAD18 to construct plasmid **2**. Thus, host cells were transformed either with plasmid **1** or **2** and afterwards co-transformed with any of the plasmids **3–19**. The α-subunits PpoC1 and PpoC19 were not included in the plasmid set due to the fact that they are lacking the consensus sequence for the coordination of the eponymous Rieske-type cluster (**C**X**H**X15-17**C**X2**H**) for Rieske non-heme iron oxygenases and in consequence are probably not catalytically active anymore (Ferraro et al. 2005).

**Fig. 4:**
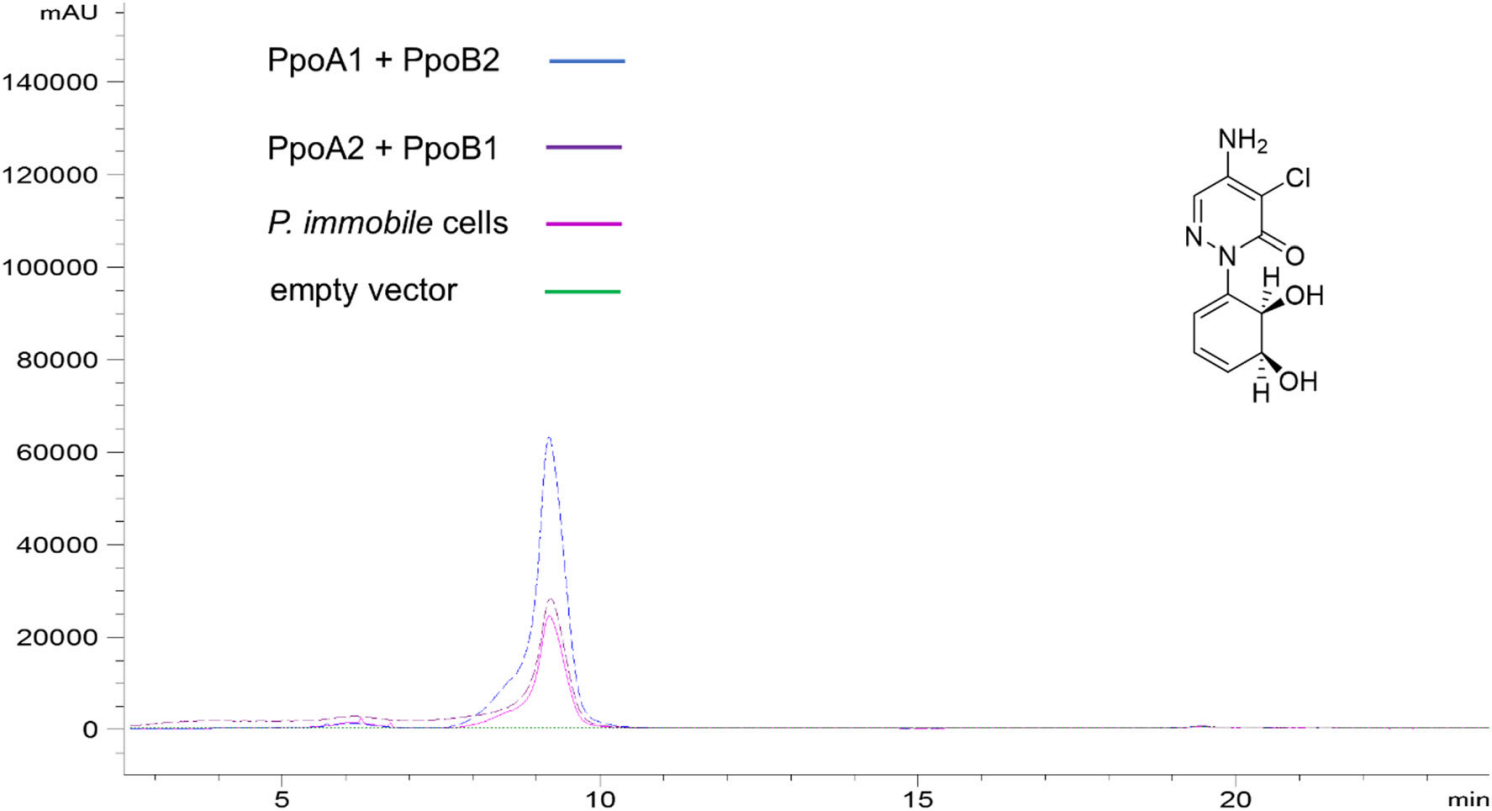
LC-MS analysis of pyrazon biotransformation. Chromatograms recorded in SIM mode (m/z: 254) with negative ionization. All *E. coli* JW5510 cells were transformed with pBAD18_PpoC11_PpoD (plasmid **12**). Blue: Biotransformations with *E. coli* JW5510 cells harboring pBAD33_PpoA1_PpoB2 (plasmid **2**). Violet: Biotransformations with *E. coli* JW5510 cells harboring pBAD33_PpoA2_PpoB1 (plasmid **1**). Pink: Biotransformations with whole *P. immobile* cells. Green: Biotransformations with *E. coli* JW5510 cells harboring empty pBAD33 vector

The expression levels of the α-subunits were too low to be detected by SDS-PAGE (see Fig. S3). To test whether any of the constructions were enabling a functional assembly of PPO, we tested all of them employing pyrazon, phenazone, L-phenylalanine, phenylacetic acid, and phenylpropanic acid as possible substrates. We analyzed biotransformations with resting cells looking for *cis*-dihydroxylated product formation. The results indicated that plasmids co-expressing *ppoc11* (plasmid **12**), along with any of the two possible redox partners (either plasmid **1** or **2**), enabled product formation, when pyrazon was used as substrate (Figs. 3b and 4).

No product standards for the expected aromatic *cis*-diol-products are commercially available. Thus, we identified the retention time and the fragmentation pattern of the pursued metabolites, employing the *cis*-dihydroxylated products generated with *P. immobile* whole cell biotransformations. Initial experiments showed very low product formation, thus we aimed to boost the activity of the identified active α-subunit PpoC11. In order to enhance product formation, we therefore investigated different conditions for protein expression, and different biotransformation temperatures. The experiments indicated that the biotransformation temperature is the parameter strongly influencing product formation (Fig. S4), while the induction time for protein expression led to have an indiscernible effect. The best identified temperature was 20 °C, both for protein expression and biotransformation reaction. By employing the improved conditions, it was also possible to detect the *cis*-dihydroxylation of phenazone with PpoC11 (Fig. S5). A summary of all performed biotransformation experiments is given in Table 3. Marked α-subunit PpoC11 showed each time product formation after co-expression with both tested redox partner pairs (plasmid **1** and **2**).

**Table 3:** List of tested substrates in biotransformations. A cross marks product formation

Substrate	α-subunit PpoC number:																		
	1	2	3	4	5	6	7	8	9	10	11	12	13	14	15	16	17	18	19
Pyrazon	nd										X								nd
Phenazone	nd										X								nd
L-phenylalanine	nd																		nd
Phenylacetic acid	nd																		nd
Phenylpropanoic acid	nd																		nd

The expression of the α-subunit PpoC11 without the β-subunit PpoD (plasmid **20**) showed no *cis*-dihydroxylation activity with either pyrazon or phenazon. Such result, along with the observation in the CN-PAGE electrophoretic separation profile, provides further evidence that a heteromeric structure (α_3_β_3_) is the quaternary structure of the oxygenase of PPO.

### Sequence alignment and homology modeling reveal a non-conserved sequence region near to the catalytic iron.

We performed an in silico protein sequence alignment, in order to compare the α-subunits encoded in the *P. immobile* genome and to examine the major differences among them. Four out of the 19 α-subunits are not shown in the alignment. The α-subunits PpoC3 and PpoC8 were excluded due to their low sequence identity (19.2–23.6 %) and smaller size (43.0 and 41.1 kDa, respectively) with respect to the other α-subunits (see Table S6). For α-subunits PpoC1 and PpoC19, the omission was based on the fact that they are lacking the consensus sequence for the coordination of the Rieske-type cluster, which is essential for the catalytic activity. The sequence identity among the remaining 15 PpoC α-subunits ranges from 63.7 to 89.0 % and differs slightly in terms of size from 51.1 ± 0.8 kDa.

The protein sequences of this 15 α-subunits mainly differ in one non-conserved sequence region. Based on the differences in this segment, the α-subunit sequences can be subdivided into two groups. The non-conserved region of group one comprises 11–23 amino acids. In addition to large deletions, the amino acid proline is frequent, indicating that the formation of secondary structures is unlikely due to the lack of a free amino group (Huang et al. 2018). The sequence identity within group one is 64.9–71.0%. In group two, the non-conserved region consists of 25–28 amino acids. At the N-terminus of this region, many polar residues like lysine, glutamic acid, and aspartic acid are present. Towards the C-terminus, small and flexible glycine residues are frequently found, up to four of them in line. The sequence identity within this group is 70.7–89.0%. To figure out the placement of this region in a three-dimensional structure, homology models with the software YASARA were generated.

We represent the three-dimensional conformation of PpoC11 from group two, which was the only α-subunit we detected *cis*-dihydroxylation of pyrazon and phenazone with in *E. coli*. The model suggests that the variable region is located near to the active site of the α-subunit. In the native trimeric structure of PpoC11, it is facing the surrounding and forming a helix structure (Fig. 6). This feature is highlighted in red in the homology model in the side and top view (Figs. 6 a, b). PpoC5 from group one was chosen as a representative of group one to depict a comparative structure of the three-dimensional conformation. In this case, the homology model predicted an unstructured loop region for the variable sequence region. Again, it is located near to the active site of the α-subunit.

## Discussion

*P. immobile* is characterized by its unique carbon sources, as it shows best growth on synthetic compounds like pyrazon and phenazone. Despite its narrow substrate spectrum, 19 different α subunits were annotated in the DNA sequence of *P. immobile* strain E (Reznicek et al., 2015). Previous to this work, the highest copy number encoding for RO α-subunits reported in literature was seven for *Sphingnobium* sp. strain PNB (Khara et al. 2014) (sequence identity < 30 % to the α-subunits from *P. immobile*). In this strain, one gene encoding an α-subunit was truncated and in another an insertion of a transposase and a resolvase disrupting the gene was identified. We also observed the presence of mobile genetic elements in *P. immobile*. In this case, the gene *ppoc11* (Scaffold 1: 1550566-1551958(+)) on the genomic DNA is clustered with a Tn3 transposase gene (Scaffold 1:1557232-1560172(+)) and a resolvase gene (Scaffold 1:1556523-1557156(-)). Additionally, the gene *ppoc11* is clustered with four other α-subunit genes sharing a close phylogenetic relationship (*ppoc10*, *ppoc12*, *ppoc13* and *ppoc14*) (see Fig. S6). Mobile genetic elements play an important role in the adaption of prokaryotes towards new environmental conditions. New phenotypic characteristics can occur, by simply introducing small changes in the nucleotide sequence during replication (Nojiri et al. 2004; Khan and Rao 2018). It is plausible that this high number of α-subunits in *P. immobile* is the result of repeated gene duplication events. Another important mobile genetic elements involved in the catabolism of prokaryotes are catabolic plasmids, which allow a fast and easy horizontal gene transfer of catabolic genes throughout bacterial populations (Pemberton and Schmidt 2001). It has been reported that genes encoding the RO elements are often encoded in operon structures, located on plasmids along with other genes of the upper-degradation-pathway (Werlen et al. 1996; Chakraborty et al. 2012). Widely studied examples are plasmid pWW0 harboring toluol dioxygenase from *Pseudomonas putida* mt-2 or the NAH7 plasmid containing naphthalene dioxygenase from *P. putida* NCIB 9816-4, (Yen et al. 1988; Burlage et al. 1989).

In the case of *P. immobile*, two α-subunits (*ppoc18* and *ppoc19*) are encoded in plasmidic DNA. It was astonishing to find that none of the α-subunit genes are located in close proximity to the electron transfer partners or the β-subunit, despite all are essential for a functional RO assembly. However, CN-PAGE analysis suggested that all expressed α-subunits in *P. immobile* form a heteromeric trimer with the β-subunit PpoD. This finding differs from all previously reported ROs in literature, since all previously annotated β-subunits always were next to a α-subunit in the genome (Chakraborty et al. 2012). Regarding the genes encoding for the electron partners, it was known that for some ROs, such as phthalate dioxygenases, the genes encoding for the electron transfer components are not always clustered with the α-subunit genes (Kweon et al. 2008). PpoA2 and PpoB1 in *P. immobile* are a combination of a ferredoxin with plant-type [2Fe-2S]_PT_ iron-sulfur cluster (pfam00111) and a glutathione reductase (pfam07992) as electron transport proteins. Such a redox partner combination is known for the carbazole 1,9a-dioxygenases CarAa and CarAaI from *Sphingomonas* sp. strain KA1 (UniProt-ID: Q84IG9_NOVK1, Q2PFA6_NOVK1). In this bacterium, the genes *fdxI*, *fdrI*, and *fdrII* are located on the 254 kb plasmid pCAR3 encoding for the ferredoxin FdxI (pfam00111) and the ferredoxin reductases FdrI and FdrII (pfam07992). The genes *fdxI* (1038-1355 (+)) and *fdrI* (1432–2655 (+)) form a cluster separate from the gene cluster car-I (4409-8642 (+)), which contains the other genes involved in carbazole degradation (Urata et al. 2006). In PPO purification studies in the 1970s, the authors also described PPO as a three-component system harboring a ferredoxin containing a plant-type iron-sulfur cluster (Sauber et al. 1977a).

In these previous studies, the instable PPO components had to be purified from the crude extract after days of fermentation. Afterwards, kinetic studies were performed with purified enzyme from *P. immobile*, which showed that the addition of detergents, such as Triton X-100 can drastically boost PPO activity, raising the question whether PPO could be a membrane protein (Rosenberg 1982). Our work provides for the first time a PPO expression platform harbored in an easy to handle model organism. This tool has the opportunity to bridge all previous studies focused on pyrazon degradation by *P. immobile*. Maybe, most of the tested α-subunits showed no product formation in *E. coli* biotransformations, because such special reaction condition requirements as reported by Rosenberg in 1982 are indeed needed. Furthermore, we consider novel studies that focus on detailed mutagenesis studies, since we have now an easy to handle expression platform and have identified the key genetic information on the PPO system.

**Fig. 5:**
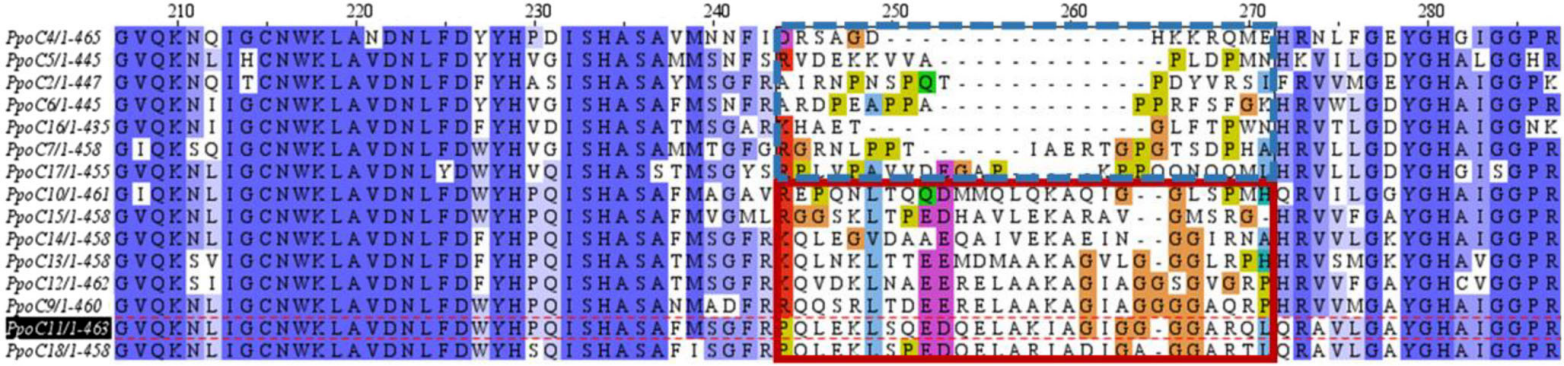
Sequence alignment of the selected set of 15 PpoC α-subunits. Classification of the different PpoC α-subunits was based on the variable segment sequence, resulting in group 1 and group 2 (blue/dash and red/solid square, respectively)

Alignments and homology models revealed that these sequence changes are mainly present in a specific region of the α-subunits, which is likely positioned near the catalytically active site in the folded protein structure (Fig. 5 c). This variable region resembles the ε-loop (E221-A238) of the well-studied naphthalene 1,2-dioxygenase (NDO) from *Pseudomonas* sp. NCIB 9816-4 (UniProt-ID: P0A110; sequence identity: 27.25–30.23 % to the α-subunits from *P. immobile* strain E). This structural feature forms a part of an access tunnel in NDO, which functions as a gatekeeper to the active site (Escalante et al. 2017) (see Fig. S7). A similar loop, also probably functioning as an gatekeeper, has been reported in the cumene dioxygenase (CDO) from *Pseudomonas fluorescens* IP01 (Dong et al. 2005), which shares a sequence identity of 33.80–37.44 % to these α-subunits. Molecular tunnels, and their flexible loop-sites, in general play important roles for enzyme engineering ( Kreß et al., 2018; Heinemann et al., 2020; Kokkonen et al., 2019). It was shown that they influence not only the specificity and selectivity of an enzyme by controlling the substrate entrance and product release. They also have a huge impact on the flexibility, and therefore the thermostability, of an enzyme (Reich et al. 2014; Nestl and Hauer 2014). *P. immobile* with its 19 different α-subunits is a remarkable example highlighting that in nature, such diversity probably means to gain new enzymatic functions. It is likely that evolution has been developing a variety of entrance tunnels and loop-areas, in order to confer broader catabolic flexibility in *P. immobile* (Campbell et al. 2016). It remains to be discovered how to tune the PPO system for the biosynthesis of targeted *cis*-diols of pharmaceutical and industrial interest.

**Fig. 6:**
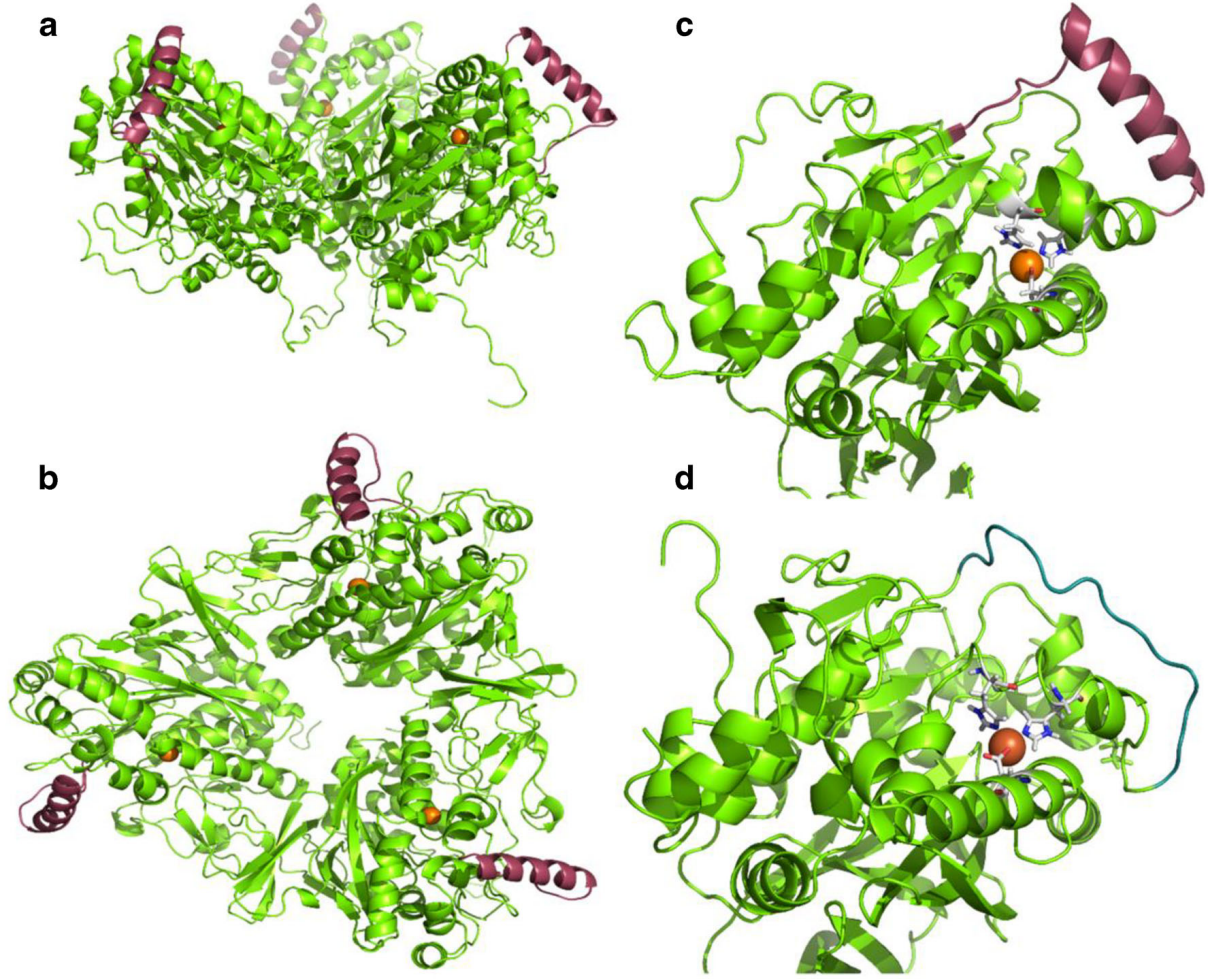
Homology models of PpoC11 and PpoC5 generated with the YASARA software. Trimer of α-subunit PpoC11 (side view) (**a**); trimer of α-subunit PpoC11 (top view) (**b**); active site of α-subunit PpoC11 (**c**); active site of α-subunit PpoC5 (**d**); variable region sequence shown in red and blue for PpoC11 and PpoC5, respectively. The mononuclear iron is shown as orange sphere. The β-subunit is not depicted

## Supplementary information

ESM 1 (PDF 1311 kb)
