# Supplementary material for: Assembly of a Rieske non-heme iron oxygenase multicomponent system from Phenylobacterium immobile E DSM 1986 enables pyrazon cis-dihydroxylation in E. coli
Source: Appl Microbiol Biotechnol. 2021 Feb 13;105(5):2003–15. doi: 10.1007/s00253-021-11129-w (PMC7907043; doi:10.1007/s00253-021-11129-w)
Supplement: Supplementary file 1 — (PDF 1311 kb) [file 253_2021_11129_MOESM1_ESM.pdf]

Assembly of a Rieske non-heme iron oxygenase multicomponent system from *Phenylobacterium immobile* E DSM 1986 enables pyrazon *cis*-dihydroxylation in *E. coli*

Andreas Hunold[1], Wendy Escobedo-Hinojosa[1], Elsa Potoudis[1], Daniela Resende[1], Per-Olof Syrén[2], Bernhard Hauer[1*]

[1]Institute of Biochemistry and Technical Biochemistry, Department of Technical Biochemistry, University of Stuttgart, Allmandring 31, 70569 Stuttgart, Germany.

[2]School of Chemical Science and Engineering, Division of Applied Physical Chemistry, KTH Royal Institute of Technology, 100 44 Stockholm, Sweden; Science for Life Laboratory, KTH Royal Institute of Technology, 171 21 Stockholm, Sweden.

*****Corresponding author:

E-mail address: bernhard.hauer@itb.uni-stuttgart.de

Phone number: +49 711 685-63193

Fax number: +49 711 685-63196

Material

Table S1 List of used bacterial strains.

Table S2 List of used plasmids.

Table S3 List of oligonucleotides used in this study.

Analytics

Table S4 LC elution gradient for the analysis of pyrazon and phenazone biotransformations.

Table S5 GC-FID temperature program for the analysis of phenylcarbonic acids biotransformations.

Oxygenases

Table S6 Identity at amino acid sequence level [%] of ROs α-subunits from *P. immobile.*

Figures

Fig. S1 Scheme of the naphthalene 1,2-dioxgenase (NDO) from *Pseudomonas* sp. NCIB 9816-4

Fig. S2 Proteome analysis results

Fig. S3 Exemplary SDS-PAGE of cells used in *in vivo* biotransformations

Fig. S4 Product formation improvement

Fig. S5 LC-MS analysis of phenazone biotransformation

Fig. S6 Genomic location mobile elements

Fig. S7 Structural comparison of NDO with the α-subunits PpoC11 and PpoC5

Bacterial strains and plasmids

**organism**	**genotype**	**characteristic**	**origin**
E. coli XL1-Blue	*rec*A1 *end*A1 *gyr*A96 *thi*-1 *hsd*R17 *sup*E44 *rel*A1 *lac* [F´ *pro*AB *lac*I^q ZΔ*M15* Tn*10* (Tet^r)]	cloning strain	Agilent Technologies Inc. Deutschland GmbH & Co. KG, Waldbronn, DE
E. coli JW5510	F– Δ(*ara*D-*ara*B)567 Δ*lac*Z4787(::*rrn*B-3) *λ*- Δ*ygj*G763::*kan rph*-1 Δ(*rha*D-*rha*B)568 *hsd*R514	expression strain	Coli Genetic Stock Center (Yale Universität)
Phenylobacterium immobile strain E	wild type	pyrazon degrader, isolated in Ecuador	DSM 1986

Table S1 List of used bacterial strains.

**plasmid**	**size [bp]**	**characteristic**	**origin**
pBAD18	6370	Amp^r<br>expression vector<br>PBAD-Promotor; ori: pBR322	Beckwith Lab
pBAD33	5352	Cm^r<br>expression vector<br>PBAD-Promotor; ori: p15A	Beckwith Lab

Table S2 List of used plasmids.

Oligonucleotides used in this study

			Sequence (5′ -> 3′)
Insert	*ppoc2*	*f*	ACCATCACCATACGGATCCGATGCGTGATCTG
		r	GATATCGGCGAACGATAGACGAGCACGCCTTCGGTCAG
Vector	pBAD33	*f*	CCCTGACCGAAGGCGTGCTCGTCTATCGTTCGCCGATATCGAAAGCCC
		r	AGCCGGACCAGATCACGCATCGGATCCGTATGGTGATGGTGAT
Insert:	*ppoc3*	*f*	GATATCGGCGAACGATAGACCTACAAGCGAGGCGACCCGTCTTCGCC
		r	ACCATCACCATACGGATCCGATGGCTCGCGGTTGCG
Vector:	pBAD33	*f*	ACGGGTCGCCTCGCTTGTAGGTCTATCGTTCGCCGATATCGAAAG
		r	GCGTCGCAACCGCGAGCCATCGGATCCGTATGGTGATGGTGAT
Insert:	*ppoc4*	*f*	ACCATCACCATACGGATCCGATGAACAGTCAGGCCGAACAG
		r	ACCATCACCATACGGATCCGATGCTCTACGAGAACGGCAAG
Vector:	pBAD33	*f*	AAGCCGGCGGCCGGATCTAGGTCTATCGTTCGCCGATATCGAAAGCC
		r	TTGCCGTTCTCGTAGAGCATCGGATCCGTATGGTGATGGTGAT
Insert:	*ppoc5*	*f*	ACCATCACCATACGGATCCGATGAAAGTCGCC
		r	GATATCGGCGAACGATAGACGACCACGCCGTC
Vector	pBAD33	*f*	CGCCTGTGGACGGCGTGGTCGTCTATCGTTCGCCGATATCGAAAGCCCG GGG
		r	AAGTTATGGGCGACTTTCATCGGATCCGTATGGTGATGGTGAT
Insert:	*ppoc6*	*f*	GATATCGGCGAACGATAGACTTAGAGCCGATCGGATGGC
		r	ACCATCACCATACGGATCCGATGCTCTACGAGAACGGCAAG
Vector:	pBAD33	*f*	TGCCATCCGATCGGCTCTAAGTCTATCGTTCGCCGATATCGAAAG
		r	TTGCCGTTCTCGTAGAGCATCGGATCCGTATGGTGATGGTGAT
Insert:	*ppoc7*	*f*	ACCATCACCATACGGATCCGATGGAGCTACGGATGCTG
		r	GATATCGGCGAACGATAGACCTAGGCTGGCGTAAGCG
Vector:	pBAD33	*f*	CGCCGCTTACGCCAGCCTAGGTCTATCGTTCGCCGATATCGAAAG
		r	TGCAGCATCCGTAGCTCCATCGGATCCGTATGGTGATGGTGAT
Insert:	*ppoc8*	*f*	ACCATCACCATACGGATCCGATGACGCAGCAGGACCAGTTCGAGAAG
		r	GATATCGGCGAACGATAGACCTAGCTGCGCCAACGCAGAACG
Vector:	pBAD33	*f*	TTCTGCGTTGGCGCAGCTAGGTCTATCGTTCGCCGATATCGAAAGCCC
		r	AACTGGTCCTGCTGCGTCATCGGATCCGTATGGTGATGGTGAT
Insert:	*ppoc9*	*f*	ACCATCACCATACGGATCCGATGCTCTATGAAAACAGAGGTCCGTT
		r	GATATCGGCGAACGATAGACCTAAGGTCGGCTATCGGCCG
Vector:	pBAD33	*f*	CGGCCGATAGCCGACCTTAGGTCTATCGTTCGCCGATATCGAAAGCC
		r	CCTCTGTTTTCATAGAGCATCGGATCCGTATGGTGATGGTGAT
Insert:	*ppoc10*	*f*	ACCATCACCATACGGATCCGATGACCATGCTTCACGACGC
		r	GATATCGGCGAACGATAGACCTAGGGCCTCGCGTC
Vector:	pBAD33	*f*	CGGCGGACGCGAGGCCCTAGGTCTATCGTTCGCCGATATCGAAAGCCC
		r	CCTCTCGTCTCGTAGAGCATCGGATCCGTATGGTGATGGTGAT
Insert	*ppoc11 + ppod*	*f*	ACCCGTTTTTTTGGGCTAGCAGGAGGATTAACCA

		r	TCTCATCCGCCAAAACAGCCATTAGAAGAAGTTGCTCAG
Vector	pBAD33	*f*	CCTGAGCAACTTCTTCTAATGGCTGTTTTGGCGGATGAG
		r	CTCTCATGGTTAATCCTCCTGCTAGCCCAAAAAAACGGGTATGG
Insert:	*ppoc12*	*f*	ACCATCACCATACGGATCCGATGCTCTACGAGACGAGAGGAC
		r	GATATCGGCGAACGATAGACCTAGGGTCGGGCGCC
Vector:	pBAD33	*f*	AGGCCGGCGCCCGACCCTAGGTCTATCGTTCGCCGATATCGAAAGC
		r	CCTCTCGTCTCGTAGAGCATCGGATCCGTATGGTGATGGTGAT
Insert:	*ppoc13*	*f*	ACCATCACCATACGGATCCGATGCTTCACGAA
		r	GATATCGGCGAACGATAGACCCGAACGCCGTC
Vector	pBAD33	*f*	CGCCCCTCGACGGCGTTCGGGTCTATCGTTCGCCGATATCGAAAGCCCGG
		r	CCCCGGATTTCGTGAAGCATCGGATCCGTATGGTGATGGTGAT
Insert:	*ppoc14*	*f*	ACCATCACCATACGGATCCGATGCTTTACGACACACAGGGC
		r	GATATCGGCGAACGATAGACTCAGACCAGATCGCCGGGAT
Vector:	pBAD33	*f*	ATCCCGGCGATCTGGTCTGAGTCTATCGTTCGCCGATATCGAAAGCCC
		r	GTCTTCATCTCGTGCAGCATCGGATCCGTATGGTGATGGTGAT
Insert:	*ppoc15*	*f*	GATATCGGCGAACGATAGACCTAGATCAGGTCGCCG
		r	ACCATCACCATACGGATCCGATGCTGCACGAGATGAAG
Vector:	pBAD33	*f*	CCCACGGCGACCTGATCTAGGTCTATCGTTCGCCGATATCGAAAG
		r	CCTTTTTCGGCGTCGACCATCGGATCCGTATGGTGATGGTGAT
Insert:	*ppoc16*	*f*	GATATCGGCGAACGATAGACCTAGACCAGTTCGCCGGC
		r	ACCATCACCATACGGATCCGATGGTCGACGCCGAAAAAG
Vector:	pBAD33	*f*	AGGCCGGCGAACTGGTCTAGGTCTATCGTTCGCCGATATCGAAAGCCC
		r	CCTTTTTCGGCGTCGACCATCGGATCCGTATGGTGATGGTGAT
Insert:	*ppoc17*	*f*	ACCATCACCATACGGATCCGATGCTGTATGACAGCACAAAG
		r	GATATCGGCGAACGATAGACTCAGACGGCGCCGACCGG
Vector:	pBAD33	*f*	CGCCGGTCGGCGCCGTCTGAGTCTATCGTTCGCCGATATCGAAAGCC
		r	TTTGTGCTGTCATACAGCATCGGATCCGTATGGTGATGGTGAT
Insert:	*ppoc18*	*f*	ACCCGTTTTTTTGGGCTAGCAGGAGGATTAACCA
		r	TCTCATCCGCCAAAACAGCCATTAGAAGAAGTTGCTCAG
Vector:	pBAD33	*f*	CCTGAGCAACTTCTTCTAATGGCTGTTTTGGCGGATGAG
		r	CTCTCATGGTTAATCCTCCTGCTAGCCCAAAAAAACGGTATGG
Insert 1:	*ppob1*	*f*	GGCTAGCAGGAGGATTAACCATGAGAGGATCTCACCATCACCATCACCATAC
		r	TCGACTCTAGACCCGGGTTATCAGGAGCCGCGGCC
Insert 2:	promotor-region pBAD	*f*	AGCCTGGCCGCGGCTCCTGATAACCCGGGTCTAGAGTCGAC
		r	TGATGGTGAGATCCTCTCATATGTAATCCTCCTGGTACCAAAAAAGAGC
Insert 3:	*ppoa2*	*f*	TGGTACCAGGAGGATTACATATGAGAGGATCTCACCATCACCATCACCATA
		r	AGCTTCTGCAGTCTAGATTATCAGGTCTCGACGAAAGCCTTC
Vector:	pBAD18	*f*	AGGCTTTCGTCGAGACCTGATAATCTAGACTGCAGAAGCT
		r	TGATGGTGAGATCCTCTCATGGTTAATCCTCCTGCTAGCCC

Insert1:	*ppob2*	*f*	GGCTAGCAGGAGGATTAACCATGGCTAACATCACCTACGTCCA
		r	TCGACTCTAGACCCGGGTTACTAGCGCTGGCTTTCCGG
Insert 2:	*ppoa1*	*f*	TGGTACCAGGAGGATTACATATGGTAGAGAAGCAGATGAGCGT
		r	AGCTTCTGCAGTCTAGATTATCAGGCGGCGACTTCTTTCA
Vector:	pBAD33	*f*	TGAAAGAAGTCGCCGCCTGATAATCTAGACTGCAGAAGCTTGG
		r	TCGACTCTAGACCCGGGTTACTAGCGCTGGCTTTCCGG
Insert:	*ppoc11* without *ppod*	*f*	GGCTAGCAGGAGGATTAACCATGAGAGGATCT
		r	CTCATCCGCCAAAACAGCC ATTAAATGAGGTCGCCAACCTTAGGC
Vector:	pBAD33	*f*	AGGTTGGCGACCTCATTTAATGGCTGTTTTGGCGGATGAGAG
		r	TGATGGTGAGATCCTCTCATGGTTAATCCTCC
Insert:	*ppod*	*f*	GGCTAGCAGGAGGATTAACCATGGTTGACGTGATTGAAAAGCCGGC
		r	CTCATCCGCCAAAACAGCCATTAGAAGAAGTT
Vector:	pBAD33	*f*	AGGTTGGCGACCTCATTTAATGGCTGTTTTGGCGGATGAGAG
		r	TGATGGTGAGATCCTCTCATGGTTAATCCTCC

Table S3 List of oligonucleotides used in this study.

Analytics

Biotransformations with **pyrazon and phenazone** were extracted with QuEChERS Universal Dispersive SPE Kit (2 mL) (Agilent, USA) according to the manufacturer's specifications. Organic phase was analysed by LC-MS using an Agilent 1260 HPLC equipped with single quad ESI-MS. The chromatographic separation of 10 µL sample was ensured with an Eclipse XDB-C8 column (2.1 x 150 mm, 3.5 µm, Agilent, USA) at 40 °C and a flow rate of 0.4 mL min^{-1}. The gradients of dH20 + 0,1 % (v/v) formic acid and methanol over time is given in Table S1.

**Time [min]**	**Ratio**	
	dH_20 + 0,1 % (V/V) formic acid [%]	methanol [%]
0	98	2
3	98	2
13	80	20
18	40	60
19	98	2
24	98	2

Table S4 LC elution gradient for the analysis of pyrazon and phenazone biotransformations.

The dihydrodiol formation was detected in SIM mode (ESI ionization; fragmentation voltage: 70 eV; drying gas flow: 12.5 L/min; nebulizer pressure: 40 psi; drying gas temperature: 325 °C; capillary voltage: + 5000 V) with positive ionization (phenazone: m/z = 223; pyrazon: m/z = 256). Biotransformations with **L-phenylalanine** were filtered (pore size: 0.45 µM) and aqueous phase was analysed by LC-MS. The chromatographic separation of 10 µL sample was ensured with an Eclipse XDB-CN (4.6 mm×150 mm, 5 µm, Agilent, USA). An isocratic gradient of H_2O-acetonitril-methanol-formic acid-25 % (w/v) ammonium hydroxide solution (23-2-2-0.005-0.005 % (v/v)) was used for 7 min at 25 °C. Aqueous phase was investigated for dihydrodiol formation in scan mode (scan m/z 80 – 400; ESI ionization; fragmentation voltage: 70 eV; drying gas flow: 12.5 L/min; nebulizer pressure: 40 psi; drying gas temperature: 325 °C; capillary voltage: + 5000 V.) L-phenylalanine was detected in SIM mode with positive ionization (m/z = 166). Biotranformations with **phenylacetic acid and phenylpropionic acid** were extracted with MTBE (+ 0.1 mM methoxyphenylacetic acid) in ratio 1:1. Organic phase was evaporated on a Genevac EZ-2 Plus Evaporator (Genevac Ltd., England) to dryness and residual dissolved in a 70 µL mixture of 50% MTBE and 50% BSTFA + TCMS (99:1). Sample were derivatised for 30 min at 70 °C. 1 µL of the samples was injected with a 1:10 split to a Shimadzu GC-2010-FID equipped with an AOC-20i auto-injector (Shimadzu, Nakagyo-ku, Japan). The samples were separated with

H_2 as carrier gas (30 cm s^{-1}, injector temperature: 250 °C) via a DB-5 column (5 % phenyl/ 95 % methyl polysiloxane) (30 m×0.25 mm×0.25 µm, Agilent, USA). The detection of the analytes was carried out using a flame ionization detector (FID, detector temperature 330 °C). The temperature program is given in Table S2.

**Start**	**Gradient**	**End**	**Hold**
		90 °C	1 min
90 °C	12 °C/min	280 °C	1 min
280 °C	65 °C/min	320 °C	3 min

Table S5 GC-FID temperature program for the analysis of phenylcarbonic acids biotransformations.

Identity at amino acid sequence level [%] of ROs α-subunits from *P. immobile*

		α subunits:																
		**2**	**4**	**5**	**6**	**7**	**9**	**10**	**11**	**12**	**13**	**14**	**15**	**16**	**17**	**18**	**3**	**8**
**α--subunits:**	2	100	65	68	70	68	69	67	68	69	68	68	68	69	68	69	22	20
	4	-	100	64	70	65	69	67	67	70	68	68	68	69	67	68	21	20
	5	-	-	100	68	66	67	67	67	68	65	66	65	70	69	68	24	21
	6	-	-	-	100	69	70	67	68	68	67	69	68	71	70	68	21	19
	7	-	-	-	-	100	66	65	64	66	65	64	64	69	71	64	22	20
	9	-	-	-	-	-	100	76	81	79	77	74	75	70	73	79	23	22
	10	-	-	-	-	-	-	100	75	74	74	71	76	69	70	75	22	21
	11	-	-	-	-	-	-	-	100	76	78	76	77	70	71	89	23	21
	12	-	-	-	-	-	-	-	-	100	77	73	74	70	70	74	22	21
	13	-	-	-	-	-	-	-	-	-	100	75	74	68	69	74	22	21
	14	-	-	-	-	-	-	-	-	-	-	100	74	68	70	77	22	21
	15	-	-	-	-	-	-	-	-	-	-	-	100	70	71	74	21	20
	16	-	-	-	-	-	-	-	-	-	-	-	-	100	71	70	23	21
	17	-	-	-	-	-	-	-	-	-	-	-	-	-	100	72	22	21
	18	-	-	-	-	-	-	-	-	-	-	-	-	-	-	100	23	21
	3	-	-	-	-	-	-	-	-	-	-	-	-	-	-	-	100	21
	8	-	-	-	-	-	-	-	-	-	-	-	-	-	-	-	-	100

Table S6 Identity at amino acid sequence level [%] of ROs α-subunits from *P. immobile.*

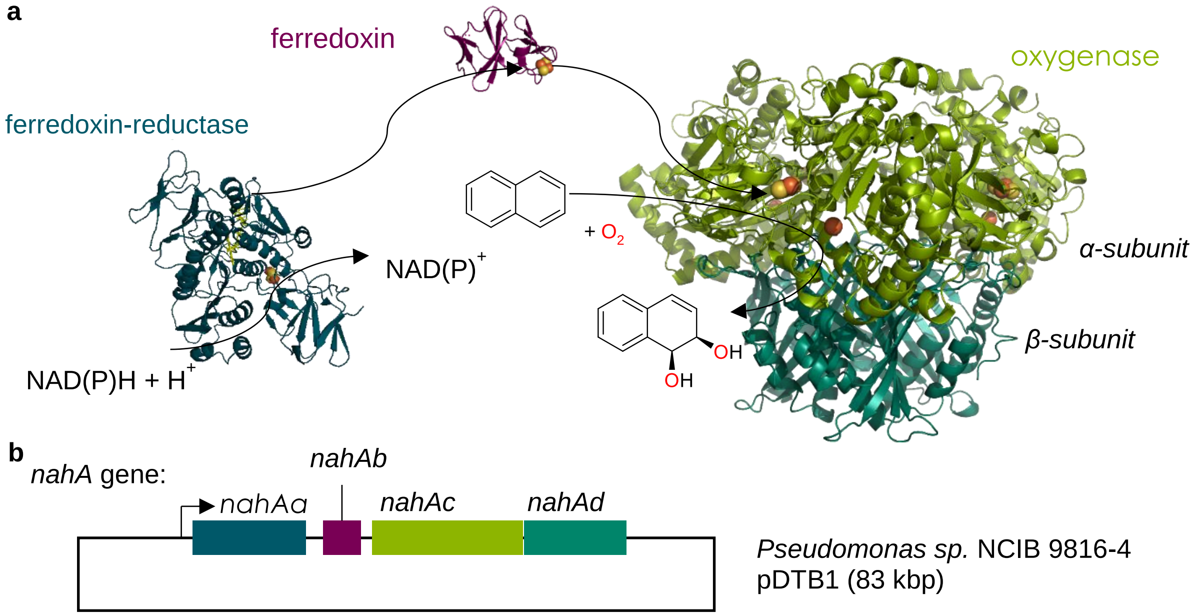

Fig. S1 Scheme of the naphthalene 1,2-dioxgenase (NDO) from *Pseudomonas* sp. NCIB 9816-4 **a**. The *nahA* gene on the catabolic plasmid pDTG1 is shown in panel **b**

Probability Legend:

- over 95%
- 80% to 94%
- 50% to 79%
- 20% to 49%
- 0% to 19%

Bio View:
Identified Proteins (17)

Identified Proteins (17)	Molecular Weight	Protein Grouping Ambiguity	E_CHL1	E_CHL2	E_CHL3	phenazone			L-phe			pyrazon			E_L_ANT1	E_L_ANT2	E_L_ANT3
						1<br>E_ANT1	2<br>E_ANT2	3<br>E_ANT3	1<br>E_50kD_1	2<br>E_50kD_2	3<br>E_50kD_3	1<br>E_L_CHL1	2<br>E_L_CHL2	3<br>E_L_CHL3			
**beta_subunit**	22 kDa		10	8	7	7	9	10	11	8	6	17	16	14	7	9	10
**Scaf2_D02**	43 kDa							3	10	7	9	6	8				3
**Scaf1_D04**	62 kDa	★							8	4	6						
**Scaf1_D07**	52 kDa	★					1		16	16	16	14	15	6		1	
**Scaf1_D08**	50 kDa	★							7	5	7						
**Scaf1_D09**	50 kDa	★		3	2	3	3	3	5	3	2				3	3	3
**Scaf1_D010**	51 kDa	★								1							
**Scaf1_D011**	41 kDa								2	7	6						
**Scaf1_D014**	52 kDa	★	2		1	2	2	6	4	1	2	5	8	4	2	2	6
**Scaf1_D015**	51 kDa	★							3		2						
**Scaf1_D016**	52 kDa	★						2	14	8	8	5	4	5			2
**Scaf1_D018**	51 kDa	★							11	7	8						
**Scaf1_D021**	51 kDa	★				1	1	3	12	9	9	8	9	5	1	1	3
**Scaf1_D022**	51 kDa	★							7	6	5		1				
**Scaf1_D023**	49 kDa	★	1	1		2	1	3	12	13	9	1	2		2	1	3
**Scaf1_D029**	51 kDa	★							5	2	5						
**plasmid_D0large _D030**	51 kDa	★	9	8	8	9	8	13	19	19	17	22	29	17	9	8	13

Fig. S2 Proteome analysis results Left lane: Name of the identified α-subunit. For nomenclature see Table S4. Numbers highlighted in green are reporting the absolute number of peptides annotated with a probability of 95%. Proteome analysis performed for all three substrates in biological triplicates

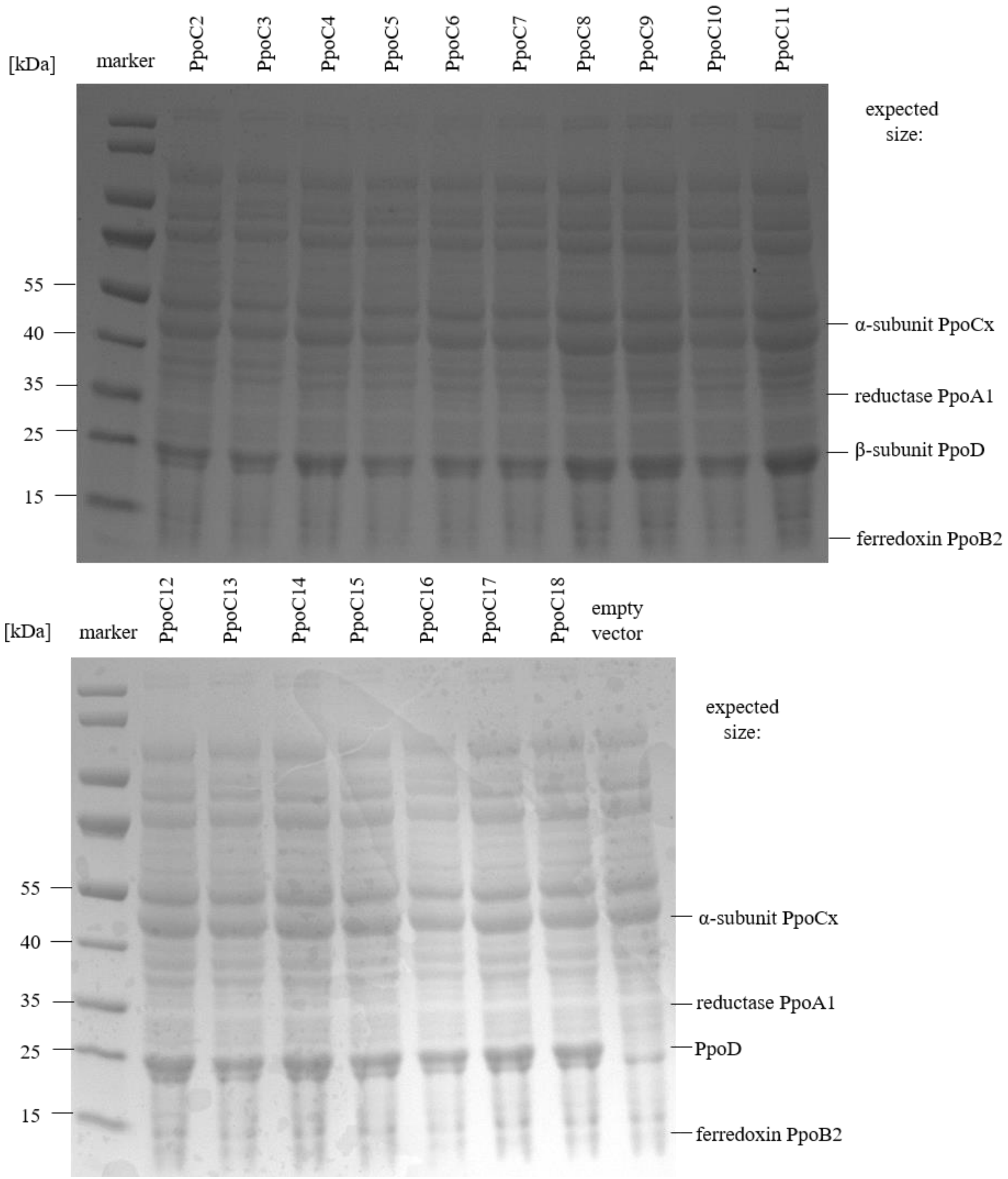

Fig. S3 Exemplary SDS-PAGE of cells used in *in vivo* biotransformations Here, *E. coli* JW5510 cells were first transformed with plasmid **2**, carrying the redox partners PpoA1 and PpoB2, and afterwards with one of the plasmids **3** – **19** (**Table 1**), carrying α-subunits PpoC2 –PpoC18 and the β-subunit PpoD. Expression performed as described in method part

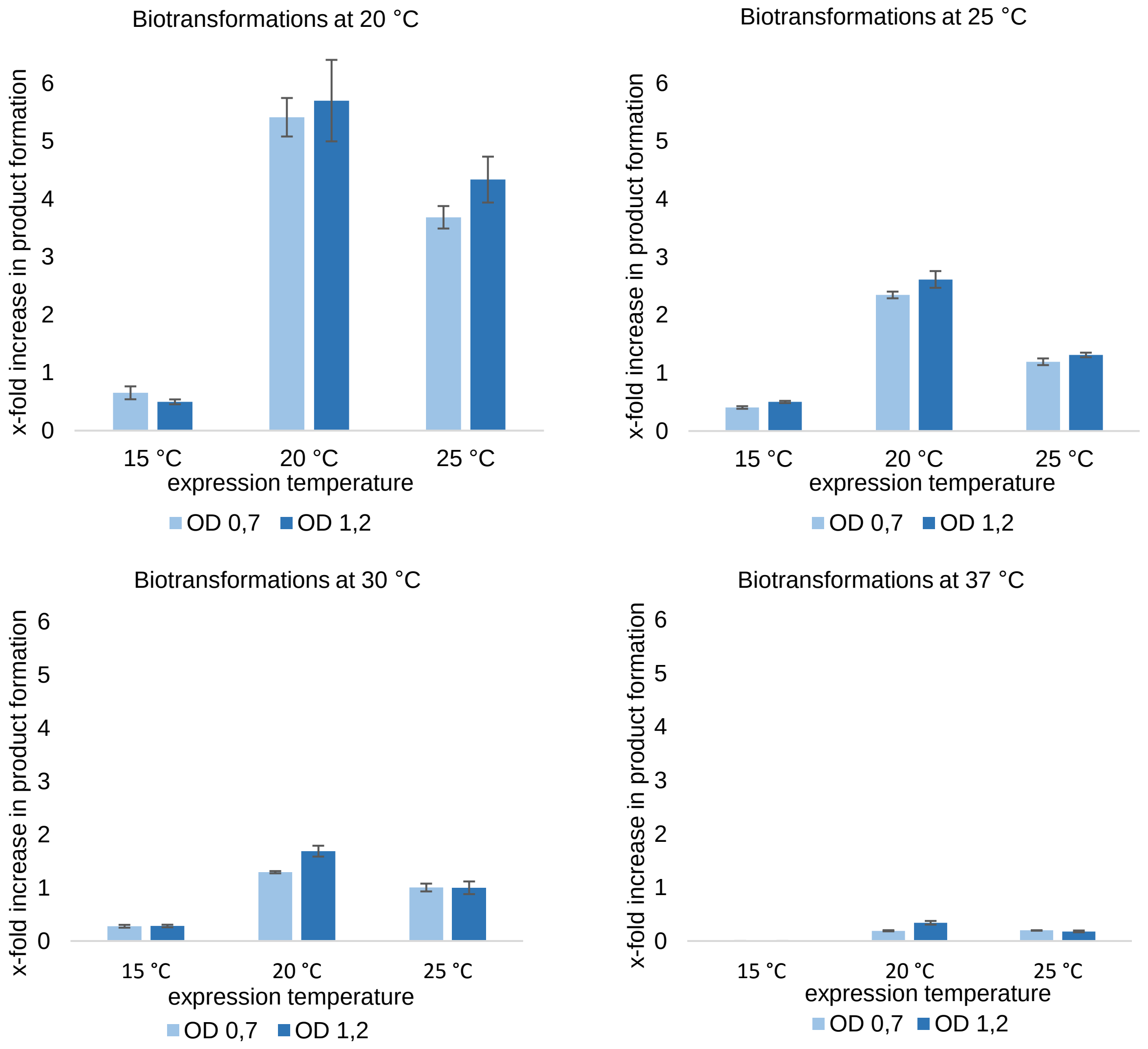

Fig. S4 Product formation improvement Altered parameters; induction OD600, expression temperature and biotransformation temperature. Initial conditions for expression: time of induction: OD 1.2; expression temperature 25 °C. Initial conditions for biotransformations were: temperature: 30 °C. All reactions were performed in biological triplicates with E coli JW5510 [pBAD33_PpoA1_PpoB2; pBAD18_PpoC11_PpoD]

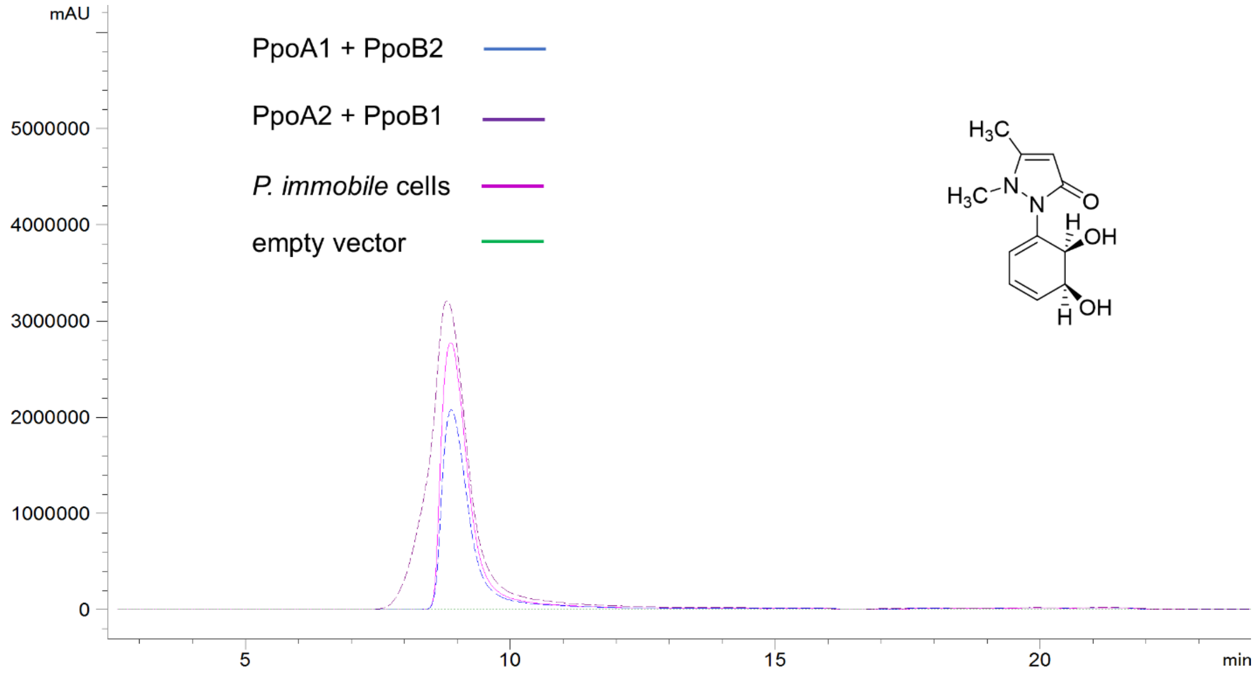

Fig. S5 LC-MS analysis of phenazone biotransformation Chromatograms recorded in SIM mode (m/z: 223) with positive ionization. All *E. coli* JW5510 cells were transformed with pBAD18_PpoC11_PpoD (plasmid **12**). Blue: Biotransformations with *E. coli* JW5510 cells harboring pBAD33_PpoA1_PpoB2 (plasmid **2**). Violet: Biotransformations with *E. coli* JW5510 cells harboring pBAD33_PpoA2_PpoB1 (plasmid **1**). Pink: Biotransformations with whole *P. immobile* cells. Green: Biotransformations with *E. coli* cells harboring empty pBAD33 vector

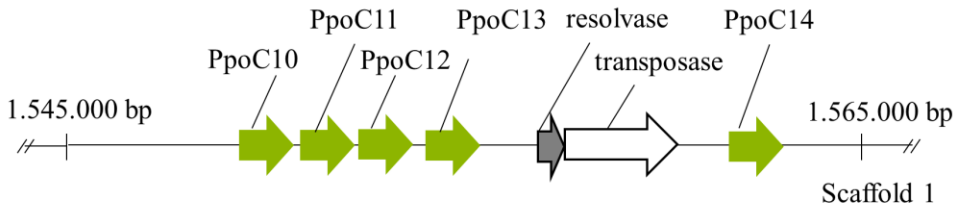

Fig. S6 Genomic location mobile elements Location of PpoC10, PpoC11, PpoC12, and PpoC13, resolvase and transposase in the *P. immobile* genome

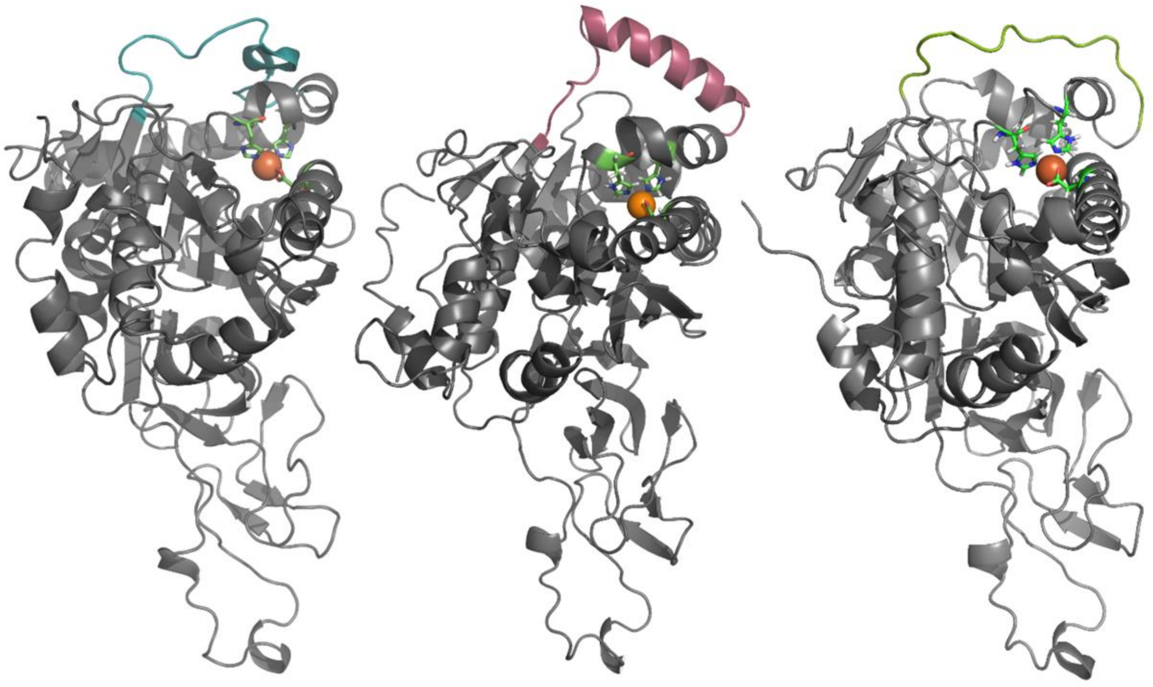

Fig. S7 Structural comparison of napthalene-1,2-dioxygenase (NDO) from *Pseudomonas* sp. NCIB 9816-4 (PDB code: 1ndo; ε-Loop in blue) with the α-subunits PpoC11 (ε-Loop in red) and PpoC5 (ε-Loop in green) from *P. immobile*. Catalytically active iron: orange. His-Asp-Triad: green sticks
